# The Intergranular Corrosion Susceptibility of Metastable Austenitic Cr–Mn–Ni–N–Cu High-Strength Stainless Steel under Various Heat Treatments

**DOI:** 10.3390/ma12091385

**Published:** 2019-04-29

**Authors:** Guangming Liu, Yuanyuan Liu, Yawen Cheng, Jin Li, Yiming Jiang

**Affiliations:** Department of Materials Science, Fudan University, Shanghai 200433, China; 16210300007@fudan.edu.cn (G.L.); 16210300002@fudan.edu.cn (Y.C.); jinli@fudan.edu.cn (J.L.)

**Keywords:** Cr–Mn–Ni–N–Cu austenitic high-strength stainless steel, intergranular corrosion, double-loop electrochemical potentiokinetic reactivation, oxalic acid etch, crystallographic orientation

## Abstract

The intergranular corrosion (IGC) behavior of a new metastable austenitic Cr–Mn–Ni–N–Cu high-strength stainless steel under various heat treatments was studied. The samples were solution treated at 1050 °C for 30 min and then aged at 600 to 900 °C for 10 to 300 min, respectively. The IGC susceptibility of aged samples was investigated using a double-loop electrochemical potentiokinetic reactivation (DL-EPR) test in a solution of 0.1 M H_2_SO_4_ and 0.002 M KSCN and the 10% oxalic acid etch. The surface morphologies of samples were characterized using optical microscopy and the scanning electron microscopy after electrochemical tests. Two time-temperature-sensitization diagrams were plotted based on the DL-EPR test and oxalic acid etching. No IGC and precipitate were observed for samples aged at 600 °C and 900 °C. For samples aged at 650 °C to 750 °C, the IGC susceptibility and the amount of precipitate both increased with the extended aging time. For samples aged at 800 °C and 850 °C, the amount of precipitate increased as the aging time was prolonged. However, only the sample aged at 800 °C for 60 min showed slight intergranular corrosion in the DL-EPR test. The IGC of the Cr–Mn–Ni–N–Cu austenitic stainless steel originated from the precipitation of Cr_23_C_6_ and Cr_2_N at the grain boundaries. The chromium-depleted zones near grain boundaries stood as the corrosion nucleation sites, but the dissolution of the weak area followed a consistent crystallographic orientation along each grain boundary.

## 1. Introduction

With the rapid development of the automotive industry, the usage of materials for automotive structures is increasing year by year. Recently, the designed service life of the electric vehicles has been significantly extended due to the introduction of replaceable batteries [1], leading to new demands for crash safety and fuel economy of the automobiles. The automotive structural materials require a combination of properties including high strength, high ductility, high corrosion resistance and lightweight to ensure the fuel economy and the extended service life [2]. Numerous studies have been concentrated on the development of promising automotive materials, including high-strength carbon steels [3], high-strength stainless steels [4,5,6], light-weight aluminum and magnesium alloys [7,8,9], and the fiber-reinforced polymers [10]. However, high-strength carbon steels and the light-weight aluminum and magnesium alloys require protective coatings to ensure corrosion resistance. The use of fiber-reinforced polymers as automotive structures is uneconomic at present [11]. New-generation high-strength stainless steel, which possesses a high product of strength and elongation (R_m_ × A, over 30 GPa%) [12], good corrosion resistance and low cost, is very promising for future automotive structures.

Austenitic stainless steels have been widely used in many areas, including the petrochemical industry, architecture, aerospace and automobile manufacturing because of their excellent properties such as high strength, good ductility, excellent formability and good corrosion resistance [13,14,15,16]. Austenitic stainless steels such as 304 and 310 are attractive for use as frame materials in automobiles [17]. However, the high cost results from the high Ni content which has limited the automotive application of these austenitic stainless steels. New attempts have been focused on developing a metastable austenitic stainless steel with mechanical properties equivalent to the traditional austenitic stainless steels at a much lower cost [18]. The expensive Ni has been replaced with low-cost Mn. Furthermore, N and Cu are simultaneously added to stabilize the austenitic phase. Nitrogen alloying can also promote the strength of stainless steel by nitrogen solution hardening [19,20]. Copper additions can reduce the needed nitrogen content and improve plasticity [21].

**Figure 1:**
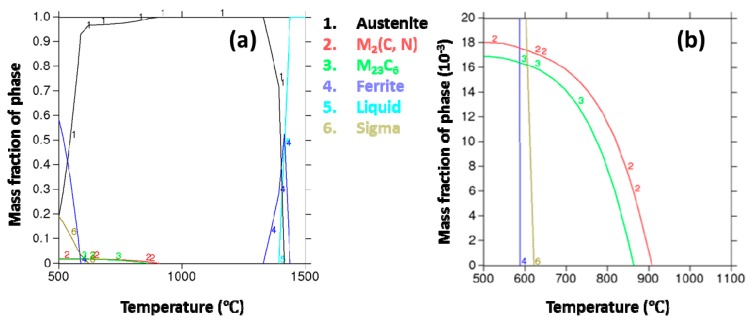
(**a**) Mass fraction of equilibrium phases for the Cr–Mn–Ni–N–Cu austenitic stainless steel determined by thermodynamic calculation; (**b**) is the partially amplified plot of (**a**). This calculation was conducted by Baosteel Co., Ltd. using Thermo-Calc with a TCFE4 database.

Recently, Baosteel Co., Ltd. in China has developed a new grade of high-strength stainless steel (13.6Cr–10.1Mn–1.19Ni–0.15N–0.85Cu) with a tensile strength greater than 1000 MPa. The martensite transformation behavior and mechanical properties of this stainless steel after cold rolling have been discussed [12]. The increase of the martensite volume fraction leads to the increase of tensile strength, and a higher volume fraction of austenite results in a larger elongation. However, the corrosion performance is the key factor to determine whether the high-strength stainless steel can meet the long-life demand of automotive applications. Austenitic stainless steels are prone to intergranular corrosion (IGC) since sensitization can easily occur after welding and heat treatments [22,23,24]. The formation of chromium-rich precipitates at grain boundaries can result in chromium-depleted zones adjacent to grain boundaries which are prone to IGC in a corrosive medium [25,26,27]. Figure 1 gives the equilibrium phase diagram obtained by thermodynamic calculation for the as-mentioned Cr–Mn–Ni–N–Cu austenitic stainless steel. At temperatures between 950 °C to 1300 °C, a pure austenite phase can be obtained. Whereas below 950 °C, chromium carbides and nitrides (e.g., M_23_C_6_, M_2_N) will precipitate and may deteriorate the corrosion resistance of steel. However, the precipitation of chromium carbides and nitrides is also controlled by the diffusion kinetics of Cr, C, and N atoms in the austenite phase [28]. The chromium depleted zone near the chromium-rich precipitates can also be repaired by the chromium atoms diffused from the inner grain at higher temperatures or for longer aging times [29,30]. The IGC susceptibility of the as-mentioned Cr–Mn–Ni–N–Cu austenitic stainless steel needs to be clarified to guide the thermomechanical and welding processes.

In this paper, the solution-treated Cr–Mn–Ni–N–Cu austenitic stainless steel samples were aged at various temperatures (600 °C to 900 °C) for different times. The microstructure evolution and IGC susceptibility were studied by morphology characterization, a double-loop electrochemical potentiokinetic reactivation (DL-EPR) test and oxalic acid etching. The IGC behavior and mechanism of the Cr–Mn–Ni–N–Cu austenitic stainless steel after various heat treatments are clarified.

## 2. Materials and Methods

### 2.1. Materials and Heat Treatments

The Cr–Mn–Ni–N–Cu metastable austenitic stainless steel studied in the present paper were provided by Baosteel Co., Ltd. (Shanghai, China) with a chemical composition shown in Table 1.

**Figure 2:**
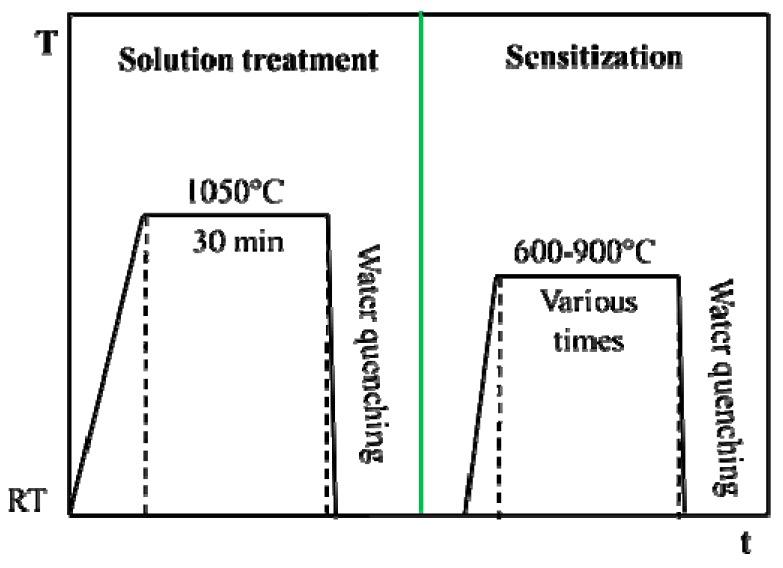
The heat treatment process of Cr–Mn–Ni–N–Cu austenitic stainless steel.

The as-received plate was cut into small samples with sizes of 12 mm × 12 mm × 2.5 mm. Then, the samples were solution-treated at 1050 °C for 30 min and water quenched to obtain a uniform dispersion of the elements. After the solution treatment, the samples were aged at 600 °C to 900 °C for 10 to 300 min, respectively. All the heat treatments were performed in a pure nitrogen atmosphere, and the detailed procedures are shown in Figure 2. Then, the samples were sealed in a mixture of epoxy and polyamide resins, grounded with SiC papers from 180 to 2000 grit, polished with the diamond paste of 2.5 μm, cleaned with ethanol, and dried in compressed air, successively. Before the electrochemical tests, the polished samples were covered by 3M tape (3M^TM^ 1600 Vinyl Electrical Tape, 3M, Saint Paul, MN, USA), leaving an exposed surface of 1 cm^2^.

### 2.2. Electrochemical Tests

The double-loop electrochemical potentiokinetic reactivation (DL-EPR) test, which comprises the cathodic polarization, monitoring of open circuit potential and the cyclic potentiodynamic polarization processes, is a fast electrochemical method for evaluating the intergranular corrosion susceptibility of stainless steels. The peak activation current density during the anodic scan (i_a_) stands for the dissolution of the whole sample surface, and the peak activation current density during the reverse scan (i_r_) represents the dissolution of weak areas like the chromium-depleted areas at the grain boundaries. R_a_ = i_r_/i_a_ × 100% was defined to evaluate the degree of IGC susceptibility of stainless steel, and a higher R_a_ value suggests the higher IGC susceptibility [31,32,33,34].

In the present work, the DL-EPR test is conducted in a solution of 0.1 M H_2_SO_4_ and 0.002 M KSCN at 30 °C using a potentiostat of CHI660e with a three-electrode cell. The treated Cr–Mn–Ni–N–Cu stainless steel sample was used as the working electrode, and a saturated calomel electrode and a platinum electrode were used as the reference electrode and the counter electrode, respectively. First, the working electrode was cathodically polarized at −0.7 V_SCE_ for 120 s to improve the reproducibility of the tests. Then, the open circuit potential was monitored for 600 s, after that the working electrode was polarized from −0.6 V_SCE_ to 0.3 V_SCE_ and back to −0.6 V_SCE_ at a scan rate of 0.1 V/min. The DL-EPR tests were repeated at least two times for each sample to ensure the data accuracy. In addition, the heat-treated samples were also etched in the 10% oxalic acid solution at 1 A/cm^2^ for 90 s [35,36,37], and the sample surfaces after etching were classified as step, dual and ditch structures according to ASTM A262-15 practice A [38].

### 2.3. Characterizations

After the DL-EPR test and the oxalic acid etching, the sample surface was cleaned with ethanol and dried in compressed air. In order to observe the precipitates, the sample aged at 700 °C for 300 min was etched in the mixture of concentrated HCl/HNO_3_ (3:1 volume fraction) for 30 s at room temperature. The surface morphologies of the samples were observed by the optical microscope (CAIKON 4XCE, CAIKON, Shanghai, China) and the scanning electron microscope (Philips, XL-30FEG, Eindhoven, The Netherlands).

## 3. Results and Discussion

### 3.1. IGC Susceptibility of Cr–Mn–Ni–N–Cu Metastable Austenitic Stainless Steel in the DL-EPR Test

**Figure 3:**
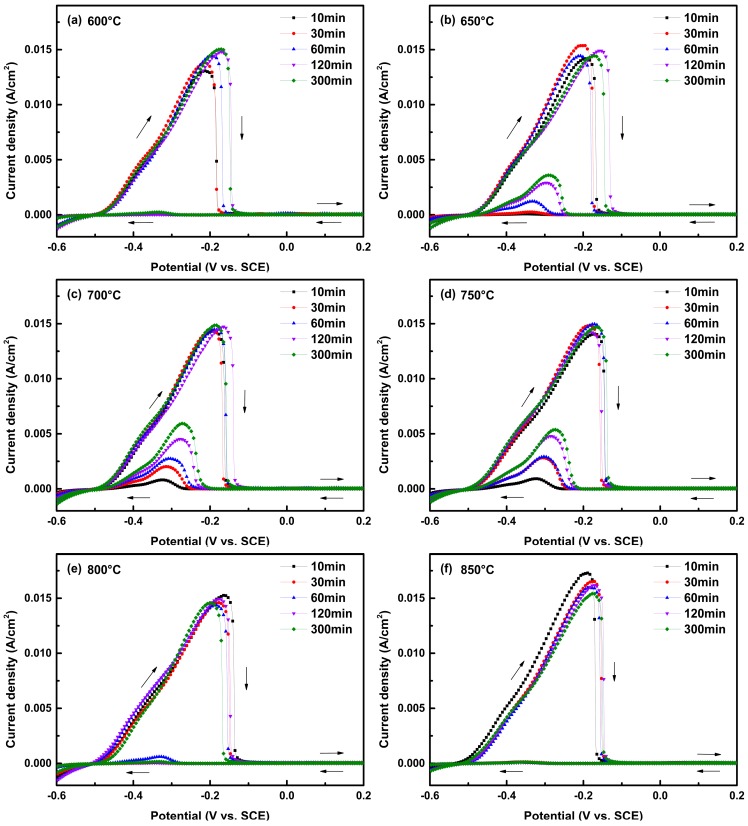

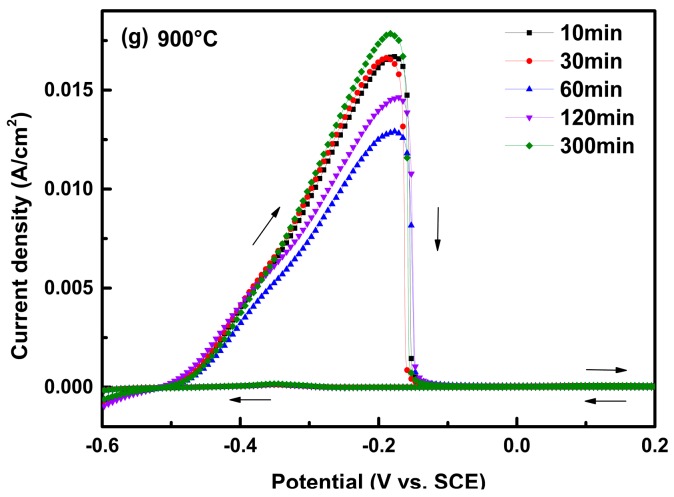
Double-loop electrochemical potentiokinetic reactivation (DL-EPR) test curves in a solution of 0.1 M H_2_SO_4_ and 0.002 M KSCN for the Cr–Mn–Ni–N–Cu austenitic stainless steel samples aged at different temperatures for various times: (**a**) 600 °C; (**b**) 650 °C; (**c**) 700 °C; (**d**) 750 °C; (**e**) 800 °C; (**f**) 850 °C; (**g**) 900 °C.

Figure 3 shows the cyclic potentiodynamic polarization curves of the Cr–Mn–Ni–N–Cu austenitic stainless steel after different heat treatments in a solution of 0.1 M H_2_SO_4_ and 0.002 M KSCN. It is worth noting that Figure 3 only displays the current densities for potentials ranged from −0.6 V_SCE_ to 0.2 V_SCE_ to better distinguish the current density peaks; although the DL-EPR test was conducted from −0.6 V_SCE_ to 0.3 V_SCE_ and back to −0.6 V_SCE_. The polarization curves of samples aged at temperatures from 600 °C to 900 °C were displayed in Figure 3a–g, respectively. For all the aging temperatures, the anodic polarization curves present similar shapes. First, the current density increased gradually with the scanning potential, and a peak current density (i_a_) of around 15 mA/cm^2^ was present near −0.2 V_SCE_ during the anodic scan, indicating the active dissolution of the whole alloy surface [39]. The current density decreased sharply to around 0 A/cm^2^ with the further increased potential, representing the growth of passive film on the sample surface. The anodic scan continued to 0.3 V_SCE_ to let the passive film grow. Then, the potential was scanned back from 0.3 V_SCE_ in the cathodic direction. The current density peak in the reverse scan was only present for samples treated at 650 °C, 700 °C, 750 °C and 800 °C, and the current density peak expanded as the aging time increased from 10 min to 300 min for samples treated at the same temperature (except 800 °C). The current density peak in the reverse scan can be associated with the dissolution of weak areas on the sensitized sample surface like the chromium-depleted zones at grain boundaries [31,40]. The absence of a current density peak in the reverse scan suggests that the samples treated at 600 °C, 850 °C and 900 °C are immune to intergranular corrosion.

**Figure 4:**
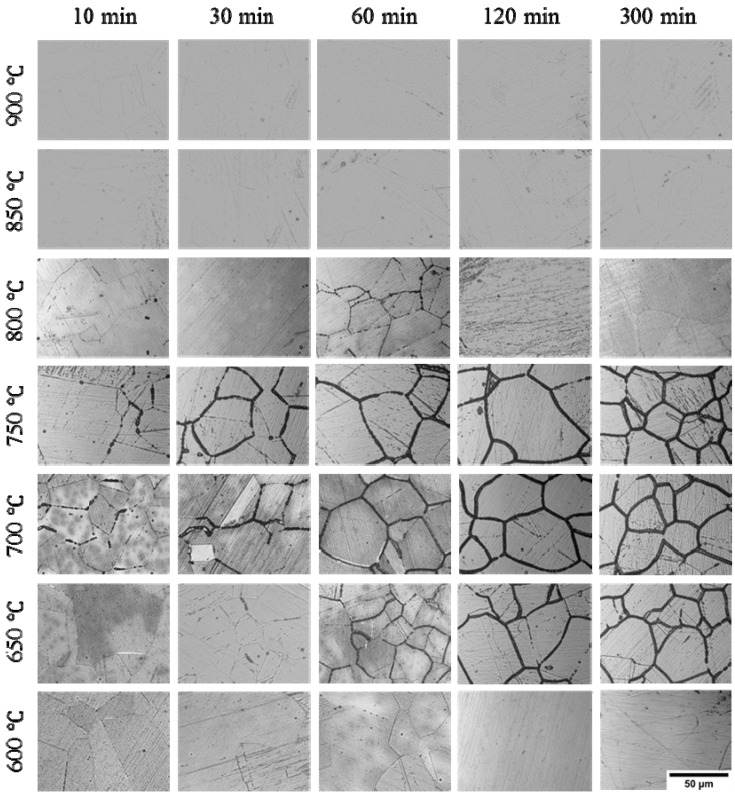
Optical micrographs of the Cr–Mn–Ni–N–Cu austenitic stainless steel samples aged under different heat treatment conditions after the DL-EPR test.

In order to quantitatively evaluate the IGC sensibility of the Cr–Mn–Ni–N–Cu austenitic stainless steel samples, the R_a_ values were calculated by i_r_/i_a_ × 100% based on the polarization curves. The R_a_ values were summarized in Table 2 as average values ± standard deviations derived from repeated tests. Although a higher R_a_ value suggests higher IGC sensibility, it is known that the inner-grain dissolution can also contribute to the current density peak in the reverse scan, leading to higher R_a_ values in the DL-EPR test [35,39]. It is necessary to evaluate the surface morphology of samples after the DL-EPR test to verify the reliability of R_a_ values and clarify the R_a_ value range that can represent different degrees of intergranular corrosion. Figure 4 shows the optical micrographs of the aged Cr–Mn–Ni–N–Cu stainless steel samples after the DL-EPR test. Despite the polishing lines, no corrosion could be observed inside the grains, suggesting that all the current density peaks in the reverse scan originated from the dissolution of grain boundaries. The derived R_a_ values can represent the IGC susceptibility of the aged Cr–Mn–Ni–N–Cu stainless steel samples. The change of corroded grain boundaries effectively matches with the trend of the derived R_a_ values.

From Table 2 and Figure 4, it could be found that the R_a_ values were all less than 2% and no IGC was observed in the optical images for samples aged at 600 °C, 850 °C and 900 °C, suggesting good IGC resistance of these samples. For samples aged at 650 °C, 700 °C and 750 °C, the R_a_ values of samples aged at the same temperature all increased with the prolonged aging time, accompanied by the gradually expanded ditches of corroded grain boundaries. When R_a_ reached the biggest value of 39.97% for the sample aged at 700 °C for 300 min, the most serious IGC morphology was observed on the sample surface, representing the highest susceptibility of IGC.

For the samples aged at 800 °C, the average R_a_ value increased to 3.55% as the aging time increased to 60 min and decreased with the extended aging time. This may suggest the repair of the chromium-depleted area near the grain boundary after 60 min. It is worth noting that the R_a_ values of samples aged for 10 and 30 min both increased with the increase of aging temperature from 600 °C to 750 °C, and then decreased with the further increase of aging temperature to 900 °C, resulting in the highest R_a_ values present at 750 °C. For samples aged for 60, 120 and 300 min, the highest R_a_ values were present at 700 °C rather than 750 °C. This agrees well with the thermodynamic calculation shown in Figure 1 that more chromium-rich phases can precipitate at 700 °C compared to 750 °C. However, it requires a longer time for the Cr, C, and N atoms to reach the grain boundary at a lower temperature [29,41].

**Figure 5:**
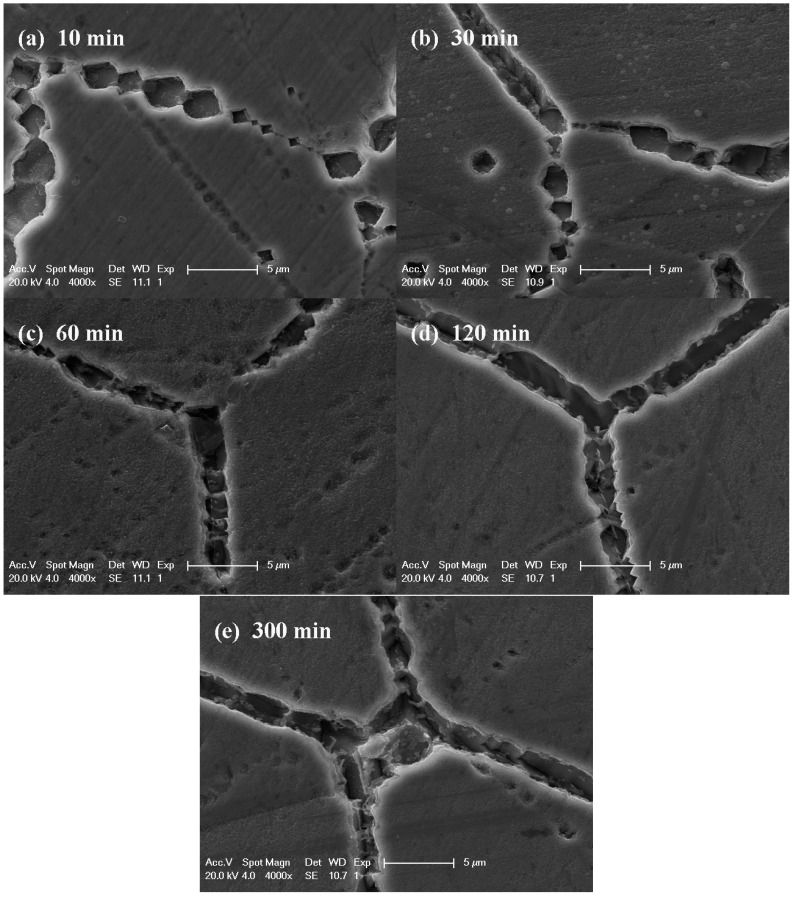
The SEM figures of Cr–Mn–Ni–N–Cu austenitic stainless steel samples treated at 750 °C for different times after the DL-EPR test: (**a**) 10 min; (**b**) 30min; (**c**) 60 min; (**d**) 120 min; (**e**) 300 min.

In order to clarify the evolution of intergranular corrosion with various heat treatments for the Cr–Mn–Ni–N–Cu austenitic stainless steel, the amplified morphologies of grain boundaries were characterized. Figure 5 shows the corroded grain boundaries after the DL-EPR test of samples aged at 750 °C for various times. As can be seen from Figure 5, discrete etch pits were observed at grain boundaries for samples aged at 750 °C for 10 and 30 min. It is worth noting that the etch pits display consistent crystallographic orientations along each grain boundary. As the aging time increased, the crystallographic etch pits grew up to continuous ditches.

**Figure 6:**
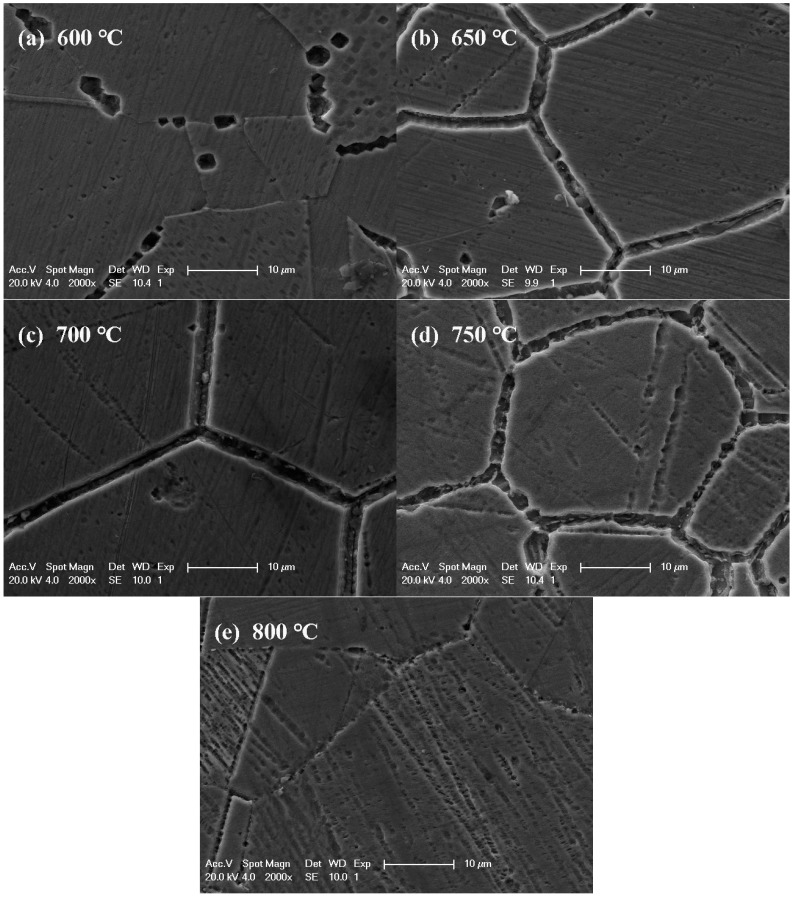
The SEM figures of Cr–Mn–Ni–N–Cu austenitic stainless steel samples treated at different temperatures for 300 min after the DL-EPR test: (**a**) 600 °C; (**b**) 650 °C; (**c**) 700 °C; (**d**) 750 °C; (**e**) 800 °C.

**Figure 7:**
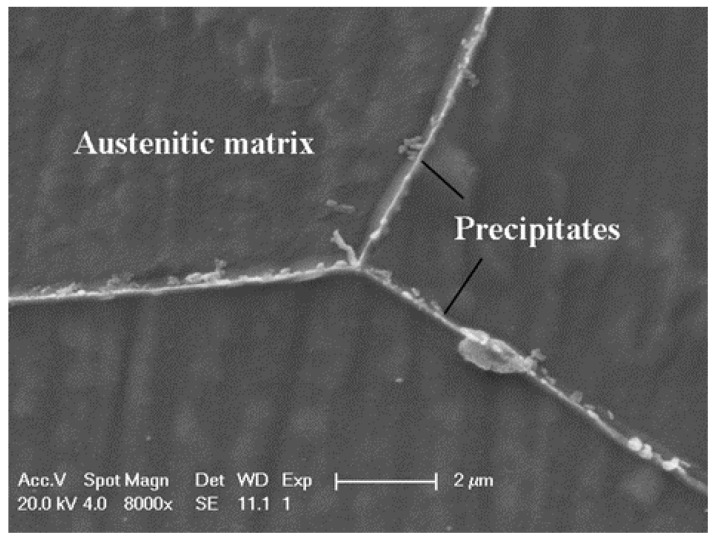
The SEM micrograph of the Cr–Mn–Ni–N–Cu austenitic stainless steel sample aged at 700 °C for 300 min after etching in the mixture of concentrated HCl/HNO_3_ (3:1 in volume fraction) for 30 s.

**Figure 8:**
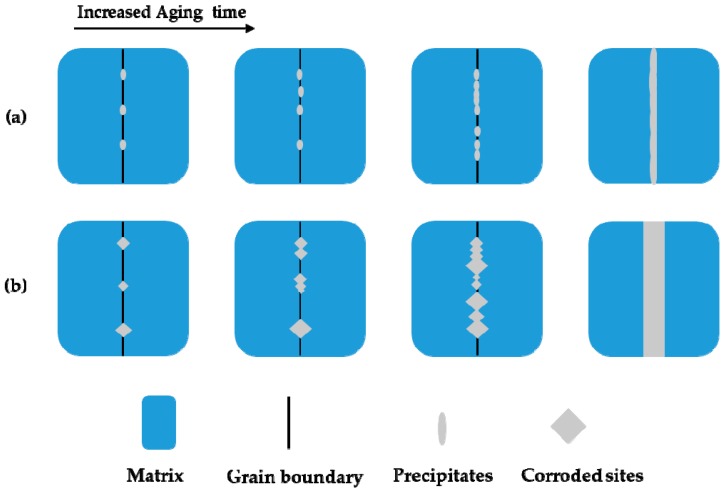
A schematic diagram for the initiation and growth of precipitates and intergranular corrosion with the extended aging time for the aged Cr–Mn–Ni–N–Cu austenitic stainless steel sample: (**a**) The growth of precipitates; (**b**) the growth of crystallographic intergranular corrosion.

Figure 6 presents the surface morphology of samples aged for 300 min at various temperatures after the DL-EPR test. The most serious IGC was observed for the sample aged at 700 °C for 300 min. No IGC occurred for the sample aged at 800 °C for 300 min. For the sample aged at 600 °C for 300 min, discrete corrosion sites with specific crystallographic orientations were also present. In order to clarify whether the crystallographic corrosion is determined by the shape of precipitates or alloy matrix, the sample aged at 700 °C for 300 min was immersed in the mixture of concentrated HCl/HNO_3_ (3:1 in volume fraction) for 30 s to etch the austenite phase matrix and expose the precipitates. From Figure 7, it can be seen that precipitates without a specific shape were nucleated and grew at the grain boundary. The crystallographic corrosion sites should be associated with the austenitic matrix near the grain boundary. A schematic diagram for the initiation and growth of precipitates and intergranular corrosion by heat treatment is shown in Figure 8 for the aged Cr–Mn–Ni–N–Cu austenitic stainless steel sample. The chromium-deficient zones near the grain boundary made the corrosion attack nucleate at the grain boundary for a shorter aging time, while the growth of these corrosion sites with the extended aging time was controlled by the crystallographic orientation. This crystallographic corrosion behavior is also common in the anodic polarization of metals and alloys, such as aluminum foil [42] and β-tin [43].

### 3.2. IGC Susceptibility of Cr–Mn–Ni–N–Cu Metastable Austenitic Stainless Steel in Oxalic Acid Etching

**Figure 9:**
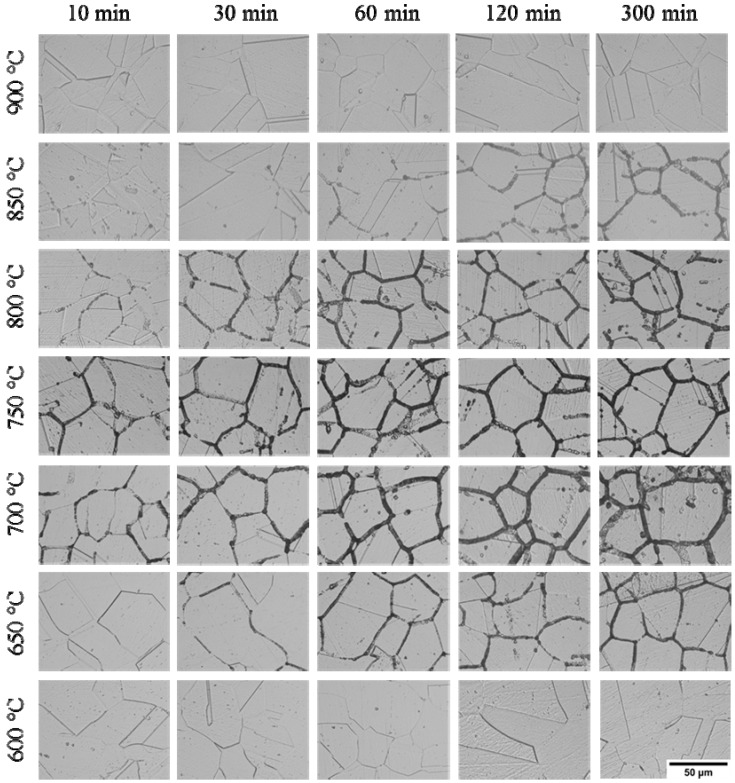
Optical micrographs of the Cr–Mn–Ni–N–Cu austenitic stainless steel samples aged under different heat treatment conditions after the 10% oxalic acid etch.

The optical micrographs of Cr–Mn–Ni–N–Cu stainless steel samples aged under different heat-treatment conditions after 10% oxalic acid etch are given in Figure 9. Coincident with the DL-EPR results, only step structures were observed for samples aged at 600 °C and 900 °C, indicating that these samples are immune to the precipitation of chromium-rich compounds and intergranular corrosion. For samples aged at 650 °C to 850 °C, only step or dual structures were present for short aging times, while ditch structures were observed for extended aging times. The IGC degree increased with the extension of aging time for samples treated at the same temperature. The most sensitive aging temperature was around 700 °C and 750 °C, which induced the most serious corrosion of grain boundaries for samples treated for the same length of time. However, it is worth noting that the observed intergranular corrosion was much more severe after oxalic acid etching compared to after the DL-EPR test for samples aged at 800 °C and 850 °C. This may be associated with the fact that the chromium-rich carbides and nitrides were dissolved with the chromium-depleted zone in the 10% oxalic acid etch, while only the chromium-depleted zone was etched in the reverse polarization of DL-EPR test [44]. At a higher aging temperature such as 800 °C and 850 °C. Although the chromium carbides or Cr_2_N can precipitate at the grain boundary and consume the nearby Cr atoms, the deficient Cr atoms could be easily replenished by the Cr atoms diffused from the inner-grain to the grain boundary [44].

### 3.3. The Time-Temperature-Sensitization (TTS) Diagram for Cr–Mn–Ni–N–Cu Metastable Austenitic Stainless Steel

**Figure 10:**
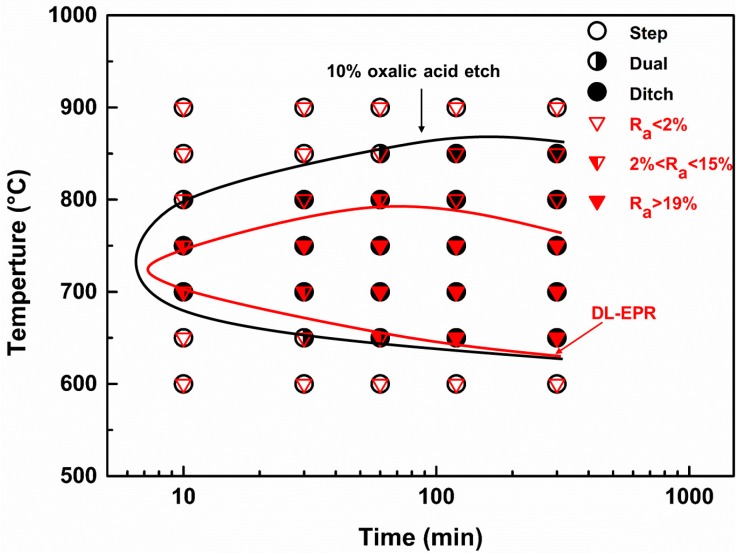
Time-temperature-sensitization (TTS) curves of the Cr–Mn–Ni–N–Cu austenitic stainless steel based on the 10% oxalic acid etch and the DL-EPR test. The red inverted triangles and black circles represent the results of DL-EPR test and oxalic acid etch, respectively.

Considering both the R_a_ values in Table 2 and the corroded surface morphologies in Figure 4, it is obvious that R_a_ values less than 2% suggest no IGC sensibility, and only steps between grains (step structure) are present on the sample surface after the DL-EPR test. For the aged samples with a R_a_ value ranged between 2% and 15%, some ditches are observed at grain boundaries, but no single grain is completely surrounded by ditches (dual structure). For the aged samples with an R_a_ value larger than 19%, more than one grain was completely surrounded by ditches (ditch structure). In Figure 10, the results of the DL-EPR test and oxalic acid etch are marked as red inverted triangles and black circles, respectively. The hollow, half empty and solid markers are corresponding to the step, dual and ditch structures, respectively. Two time-temperature-sensitization (TTS) diagrams were drawn based on the results of the DL-EPR test and oxalic acid etch. An evident small C curve was derived from the DL-EPR test. The samples aged at 650°C to 750 °C have a high tendency to intergranular corrosion, and the nose temperatures of the two C curves were about 700 °C to 750 °C.

## 4. Conclusions

In this paper, the IGC of the Cr–Mn–Ni–N–Cu austenitic stainless steel aged at 600 °C to 900 °C for 10 to 300 min was investigated using DL-EPR test and oxalic acid etch. The main conclusions are listed as follows:

(1) The results of DL-EPR test and oxalic acid etch for the aged Cr–Mn–Ni–N–Cu austenitic stainless steel indicated that IGC would occur for samples aged at about 650 °C to 750 °C, and the IGC susceptibility became more severe as the aging time increased. For samples aged at 800 °C, the IGC susceptibility increased first and then decreased with the increased aging time, suggesting the quick repair of chromium-depleted zones after longer aging at 800 °C.
(2) The nose sensitization-temperature of the Cr–Mn–Ni–N–Cu austenitic stainless steel was about 700°C to 750 °C, suggesting that the IGC could occur in a short time at about 700 °C to 750 °C.
(3) The precipitates stood as nucleation sites for the intergranular corrosion attack of the Cr–Mn–Ni–N–Cu austenitic stainless steel. However, the growth of these corrosion sites with the extended aging time obeyed consistent crystallographic orientation along the grain boundary.

## Figures and Tables

**Table 1:** The chemical composition (wt %) of Cr–Mn–Ni–N–Cu austenitic stainless steel.

Element	C	N	S	Si	Mn	P	Cr	Ni	Cu	Fe
wt %	0.08	0.15	0.002	0.30	10.12	0.04	13.62	1.19	0.85	Bal.

**Table 2:** R_a_ values (%) of the Cr–Mn–Ni–N–Cu austenitic stainless steel samples under different heat treatment conditions after the DL-EPR test.

Temperature (°C)	R_a_ value (%)				
	10 min	30 min	60 min	120 min	300 min
900	0.70 ± 0.11	0.61 ± 0.14	0.80 ± 0.24	0.85 ± 0.17	0.75 ± 0.14
850	0.70 ± 0.01	0.76 ± 0.07	0.56 ± 0.10	0.33 ± 0.14	0.61 ± 0.12
800	0.47 ± 0.05	0.75 ± 0.17	3.55 ± 0.63	0.39 ± 0.08	1.90 ± 0.82
750	6.48 ± 0.05	19.87 ± 0.94	19.36 ± 0.11	31.30 ± 2.14	34.89 ± 1.64
700	5.97 ± 0.32	14.98 ± 0.95	20.00 ± 1.10	33.12 ± 2.37	39.97 ± 0.10
650	0.39 ± 0.13	1.68 ± 0.05	8.74 ± 0.27	19.48 ± 0.02	25.40 ± 0.47
600	0.42 ± 0.11	0.22 ± 0.05	0.22 ± 0.05	0.13 ± 0.04	1.73 ± 0.19
